# Atosiban versus nifedipine for the management of preterm labor: a systematic review and meta-analysis of randomized controlled trials

**DOI:** 10.61622/rbgo/2026rbgo55

**Published:** 2026-08-20

**Authors:** Alessandro Pascon, Ana Júlia Fumes, Nícolas Hammad Rüdinger, Ricardo Filipe Alves da Costa, Lucas Borges Pereira, Luiz Augusto Beltramin Martins

**Affiliations:** 1 Faculdade de Ciências da Saúde de Barretos Doutor Paulo Prata São Paulo Brazil Faculdade de Ciências da Saúde de Barretos Doutor Paulo Prata, Barretos, São Paulo, Brazil.; 2 Hospital de Amor Centro de Pesquisa em Oncologia Molecular São Paulo Brazil Centro de Pesquisa em Oncologia Molecular, Hospital de Amor, Barretos, São Paulo, Brazil.

**Keywords:** Atosiban, Nifedipine, Preterm labor, Tocolysis, Neonatal intensive care unit

## Abstract

**Objective:**

To compare the efficacy, maternal safety, and neonatal outcomes of atosiban versus nifedipine in the management of preterm labor, with particular attention to neonatal intensive care unit (NICU) admission rates.

**Data sources:**

PubMed, EBSCOHost, ClinicalTrials.gov and the World Health Organization International Clinical Trials Registry Platform (ICTRP), and the Cochrane Central Register of Controlled Trials were systematically searched up to May 25, 2025. No language restrictions were applied.

**Study selection:**

Randomized controlled trials (RCTs) comparing atosiban and nifedipine in women with clinically diagnosed preterm labor were included, provided they reported at least one outcome related to pregnancy prolongation. Six RCTs comprising 1,070 patients met inclusion criteria.

**Data collection:**

Two reviewers independently extracted data on efficacy, maternal adverse effects, and neonatal outcomes using a standardized form. Risk of bias was assessed with the Cochrane Risk of Bias 2 (RoB 2) tool.

**Data synthesis:**

Pooled analyses showed no significant difference between atosiban and nifedipine in delaying delivery for 48 hours (risk ratio [RR] 1.09; 95% confidence interval [CI] 0.97-1.23) or 7 days (RR 1.04; 95% CI 0.89-1.22). Atosiban was associated with a significantly lower risk of maternal headache, hypotension, and tachycardia. Atosiban was associated with a non-statistically significant trend toward higher NICU admission (RR 1.36; 95% CI 0.97-1.94; p=0.079), an estimate derived from only two RCTs with substantial heterogeneity (I^2^=69.8%) and warranting cautious interpretation. No significant differences were observed for neonatal death, sepsis, or hemorrhagic disease.

**Conclusion:**

Atosiban and nifedipine offer comparable tocolytic efficacy. Atosiban carries a more favorable maternal safety profile but shows a non-significant trend toward higher NICU admission warranting confirmation in larger trials. Tocolytic selection should therefore be individualized, weighing maternal tolerability against this uncertain neonatal signal.

**Systematic Review Registration:** PROSPERO CRD420251065426

## Introduction

Preterm labor, defined as the onset of regular uterine contractions leading to cervical changes before 37 weeks of gestation, remains one of the foremost contributors to neonatal morbidity and mortality worldwide.^(1-3)^ In 2020 alone, approximately 13.4 million babies were born preterm, representing one in every ten live births.^(2)^ The stagnation of these rates over the past decade has led the World Health Organization to characterize preterm birth as a "silent emergency."^(4-6)^ As the leading cause of neonatal death globally, accounting for approximately 35% of annual neonatal mortality, the clinical management of preterm labor remains a central priority in obstetric care.^(5)^

Tocolytic therapy aims primarily to delay delivery long enough to allow for antenatal corticosteroid administration and, when necessary, maternal transfer to a facility capable of caring for a preterm neonate. This 48-hour window is considered the critical benchmark for tocolytic success, as it is sufficient to optimize fetal lung maturation.^(7)^ Among the tocolytic agents in widespread clinical use, atosiban and nifedipine represent the two most commonly compared options. Atosiban is a competitive oxytocin receptor antagonist that reduces myometrial contractility by limiting intracellular calcium release,^(8,9)^ while nifedipine is a calcium channel blocker that achieves uterine relaxation through inhibition of calcium ion influx.^(10,11)^ Nifedipine offers practical advantages in terms of oral bioavailability and substantially lower cost, though it carries recognized risks of maternal hypotension and reflex tachycardia.

The clinical choice between these agents remains unsettled, with individual trials and prior systematic reviews reporting conflicting results regarding pregnancy prolongation, maternal safety, and neonatal outcomes.^(12)^ Of particular concern, emerging data suggest that atosiban may be associated with higher rates of NICU admission, a finding with direct implications for neonatal resource utilization and healthcare costs. This updated systematic review and meta-analysis was conducted to synthesize the best available evidence on the comparative efficacy and safety of atosiban versus nifedipine, with focused attention on neonatal outcomes. Although prior systematic reviews have compared these agents, several important evidence gaps remain. The most recent aggregate-level meta-analysis (Ali et al., 2019)^(13)^ did not pool neonatal intensive care unit (NICU) admission data and predated the publication of Singh et al. (2024)^(14)^, the most recent randomized trial on this topic; furthermore, that review reported only a non-significant trend toward reduced maternal hypotension with atosiban, an endpoint for which the present analysis now provides statistically robust evidence. The individual participant data meta-analysis by van Winden et al. (2022)^(15)^ employed distinct statistical methodology and did not include trials published after 2016. The present meta-analysis therefore addresses these gaps by: (1) incorporating the most recent RCT (Singh et al., 2024)^(14)^; (2) systematically quantifying NICU admission as a primary neonatal outcome with pooled risk ratios; (3) providing strengthened evidence on maternal hypotension; and (4) reporting in accordance with PRISMA 2020 methodology with a pre-registered protocol, offering an updated synthesis relevant to contemporary clinical and health-economic decision-making, including in settings where nifedipine cost advantages are of particular relevance.

## Methods

### Study design and registration

This systematic review and meta-analysis was conducted and reported in accordance with the Preferred Reporting Items for Systematic Reviews and Meta-Analyses (PRISMA) 2020 guidelines.^(16)^ The research question followed the PICO framework: population, pregnant women with a clinical diagnosis of preterm labor; intervention, atosiban; comparator, nifedipine; outcomes, delivery delay, maternal adverse effects, and neonatal outcomes. As this review was conducted exclusively on previously published, de-identified data, no institutional ethics committee approval was required. The protocol was registered in PROSPERO (CRD420251065426).

### Eligibility criteria

Eligible studies were randomized controlled trials (RCTs) that directly compared atosiban and nifedipine as primary tocolytic agents in pregnant women with a clinical diagnosis of preterm labor, and that reported at least one pre-specified efficacy or safety outcome. Adverse effects were pooled only when reported by three or more independent trials. No restriction was applied regarding institutional setting (public, private, or academic hospitals were all eligible) or geographic region. Databases were searched from inception through May 25, 2025; no start-date restriction was applied, as no trials on this comparison were published prior to 2000. No language restrictions were applied to the database searches. Studies were excluded if they: (1) were not RCTs; (2) did not directly compare atosiban with nifedipine as primary agents; (3) did not report at least one pre-specified efficacy or safety outcome; (4) did not provide sufficient data for effect-size calculation and from which complete data could not be retrieved from study authors; or (5) were conference abstracts without sufficient methodological detail.

### Search strategy

PubMed, EBSCOHost, ClinicalTrials.gov, World Health Organization International Clinical Trials Registry Platform (ICTRP), and the Cochrane Central Register of Controlled Trials were searched on May 25, 2025, using the terms "atosiban," "nifedipine," "preterm labor," "premature labor," and "PTL." The ICTRP search yielded no records. In total, five databases were searched. The full search strategy is detailed in Supplementary Material (Table 1S). In addition, reference lists of all included studies, prior systematic reviews, and relevant meta-analyses were manually screened for additional eligible reports not captured by the electronic database searches.

### Study selection

All retrieved records were imported into Zotero for deduplication. Two reviewers (A.P.F. and A.J.F.) independently screened titles and abstracts, followed by full-text assessment of potentially eligible studies. Disagreements were resolved by discussion, with arbitration by a third reviewer (N.H.R.) when consensus could not be reached.

### Data extraction

Data were extracted independently by two reviewers using a standardized form capturing study characteristics, population demographics (age, gestational age, parity, cervical dilation), drug regimens, and all relevant efficacy and safety endpoints. Primary efficacy outcomes were delay of labor for at least 48 hours and at least 7 days. Maternal safety endpoints included hypotension, headache, tachycardia, and palpitations. Neonatal outcomes included NICU admission, neonatal death, sepsis, intraventricular hemorrhage, and hemorrhagic disease. Disagreements were resolved by consensus.

### Risk of bias and publication bias assessment

Methodological quality was independently assessed using the Cochrane Risk of Bias 2 (RoB 2) tool,^(17)^ evaluating domains of randomization, deviations from intended interventions, missing outcome data, measurement of outcomes, and selective reporting. Each domain was rated low, some concerns, or high risk of bias. Publication bias for the primary efficacy outcome was assessed by visual inspection of a funnel plot; formal statistical testing was not performed given the small number of included studies.

### Data synthesis

All six included RCTs were potentially eligible for each planned synthesis. A given trial was incorporated into a specific outcome's pooled analysis only if it reported that outcome with sufficient numerical data (event counts and denominators) to calculate an effect size. As pre-specified in the PROSPERO protocol, binary outcomes were pooled when three or more independent trials reported the outcome; when data were available from fewer than three trials, results are presented narratively. This threshold was applied consistently for both efficacy and adverse-effect outcomes. Studies contributing to each individual synthesis are identified in the corresponding results sub-section.

### Statistical analysis

All analyses were performed in R version 4.2.2 using the "meta" package, with two-tailed tests and a significance threshold of p<0.05. Binary outcomes were expressed as risk ratios (RRs) with 95% confidence intervals (CIs), pooled using the Mantel-Haenszel method. Between-study variance (τ^2^) was estimated with the Paule-Mandel estimator. Heterogeneity was quantified by Cochran's Q test and the I^2^ statistic, with significance defined as p≤0.10 or I^2^>25%. Depending on the degree of heterogeneity, fixed-effect or random-effects models were applied. Leave-one-out sensitivity analyses were conducted to assess the influence of individual studies on pooled estimates.

## Results

### Study selection and characteristics

The initial search yielded 201 records. After deduplication and screening, 6 studies comprising 1,070 patients met inclusion criteria (Figure 1). Of these, 506 (47.3%) patients received atosiban and 564 (52.7%) received nifedipine. Study characteristics are summarized in Chart 1. ^(14,18-22)^ Full details of drug regimens and patient characteristics are further provided in Supplementary Material (Table 2S).

**Figure 1:**
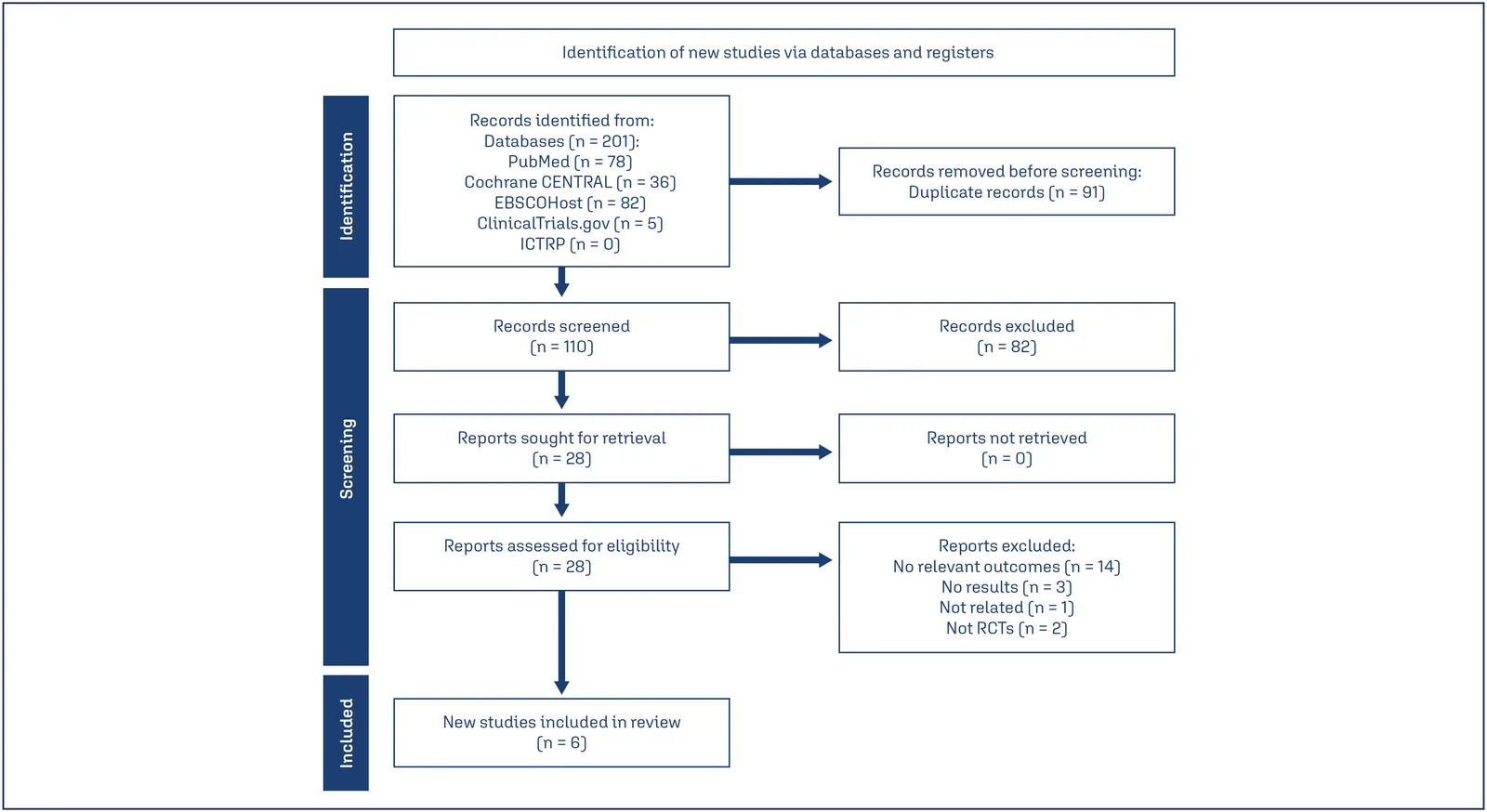
Flow diagram of study screening and selection (PRISMA 2020).

**Chart 1:** Characteristics of included studies

Study, Year, Country	Population	Design	Gestational Age (Mean or Range)	Parity	n (Ato / Nife)
Kashanian et al., 2005; Iran^(18)^	GA 26–34 wk; ≥4 contr/20 min; dilation ≥1 cm; effacement ≥50%	RCT	26–34 wk (range)	N/A	40 / 40
Al-Omari et al., 2006; Iraq^(19)^	Women 18–35 y; GA 24–35 wk; PTL per Moutquin criteria	RCT	30.84±2.86 (A) 30.84±2.82 (N)	A: 35.5% nullip N: 25% nullip	31 / 31
Salim et al., 2012; Israel^(20)^	Women with PTL; intact membranes; GA 24+0–33+6 wk	RCT	31.1 (24.1–33.8) (A) 31.8 (25.0–33.8) (N)	A: 35.7% nullip N: 49.3% nullip	70 / 75
van Vliet et al., 2016 (APOSTEL III); Netherlands^(21)^	Women ≥18 y; threatened PTL; GA 25+0–34+0 wk; singleton or multiple	Multi- center RCT	30.3 (28.1–31.7) (A) 30.3 (28.4–32.1) (N)	A: 67% nullip N: 65% nullip	255 / 248
Singh et al., 2024; India^(14)^	Women >18 y; GA 25–34 wk; dilation >1 & 3 cm; effacement >80%; intact membranes	RCT	25–34 wk (range)	N/A	60 / 70
Madkour et al., 2013; UAE^(22)^	Women 20–35 y; singleton; GA 26–34 wk; dilation 0–3 cm	RCT	26–34 wk (range)	A: 40 primigravidae; N: 38 primigravidae	50 / 50

A: atosiban group; GA: gestational age; N:nifedipine group; N/A: not available; nullip: nulliparous; PTL: preterm labor; RCT: randomized controlled trial; wk: weeks.

Full drug regimen details are provided in Supplementary Material (Table 2S).

### Delay of preterm labor by 48 hours

Five RCTs^(14,18-21)^ evaluated the efficacy of atosiban versus nifedipine in delaying delivery for at least 48 hours, encompassing 456 women in the atosiban arm and 464 in the nifedipine arm. Successful delay was achieved in 326/456 (71.5%) with atosiban and 315/464 (67.9%) with nifedipine, yielding an RR of 1.09 (95% CI 0.97-1.23; p=0.140), with moderate heterogeneity (I^2^=48.5%) (Figure 2).

**Figure 2:**
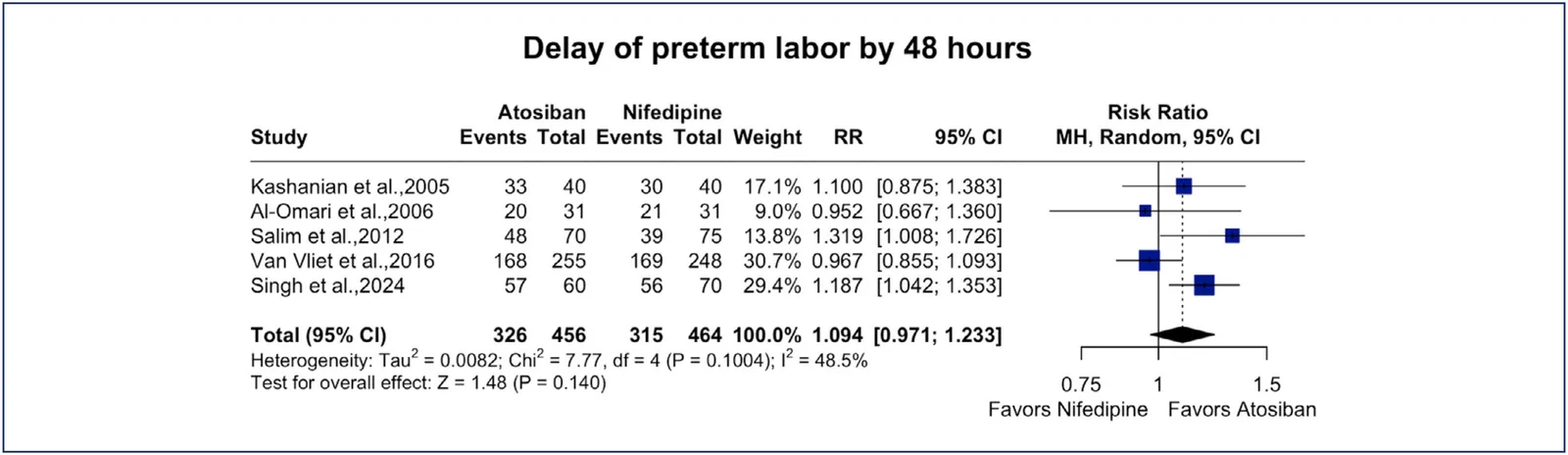
Forest plot showing pooled risk ratios for pregnancy prolongation of 48 hours or more

### Delay of preterm labor by 7 days

Five studies^(18-22)^ contributed data on 7-day delay (446 atosiban, 444 nifedipine). Successful delay for at least 7 days was reported in 236/446 (52.9%) with atosiban and 236/444 (53.2%) with nifedipine (RR 1.04; 95% CI 0.89-1.22; p=0.641; I^2^=28.7%), with no statistically significant difference between groups (Figure 3).

**Figure 3:**
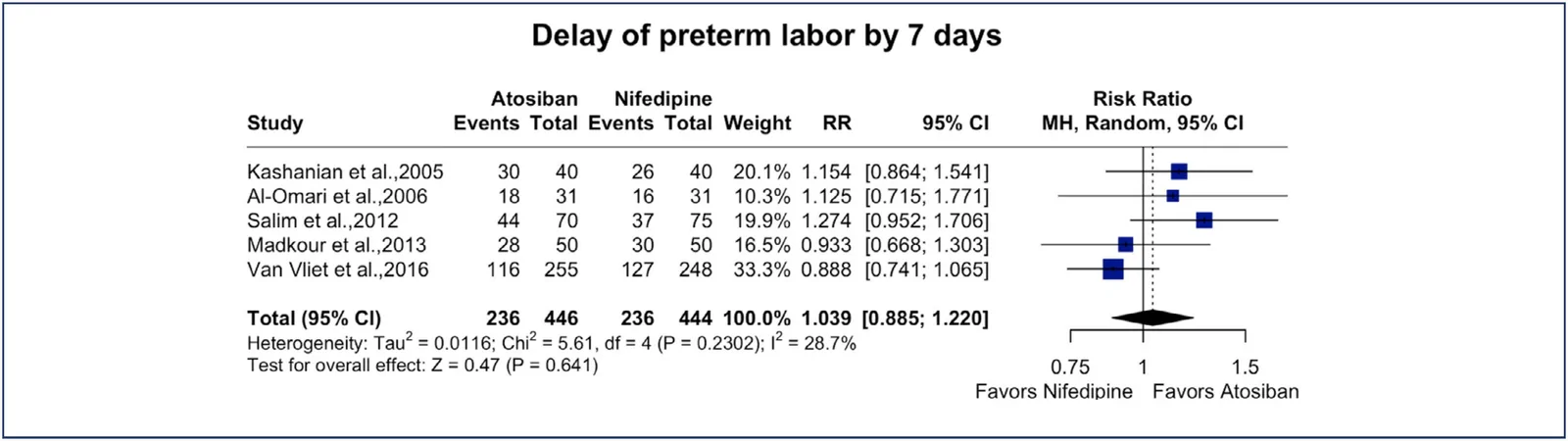
Forest plot showing pooled risk ratios for pregnancy prolongation of 7 days or more.

### Maternal safety outcomes

Across five RCTs^(14,18-20,22)^ (n=517), atosiban was associated with a significantly lower incidence of maternal headache (RR 0.45; 95% CI 0.24-0.85; p=0.014; I^2^=0%) (Figure 4). Pooled data from five studies^(14,18-21)^ (n=920) also demonstrated a markedly reduced risk of hypotension with atosiban (RR 0.24; 95% CI 0.11-0.50; p<0.001; I^2^=27%) (Figure 5). Similarly, the risk of tachycardia was significantly lower in the atosiban group across four trials^(14,18-20)^ (n=417; RR 0.40; 95% CI 0.18-0.88; p=0.022; I^2^=0%) (Figure 6). For maternal palpitations, pooled analysis of four studies^(18-20,22)^ (n=387) did not demonstrate a statistically significant difference (RR 0.36; 95% CI 0.10-1.38; p=0.14; I^2^=39%), though the point estimate numerically favored atosiban (Figure 7).

**Figure 4:**
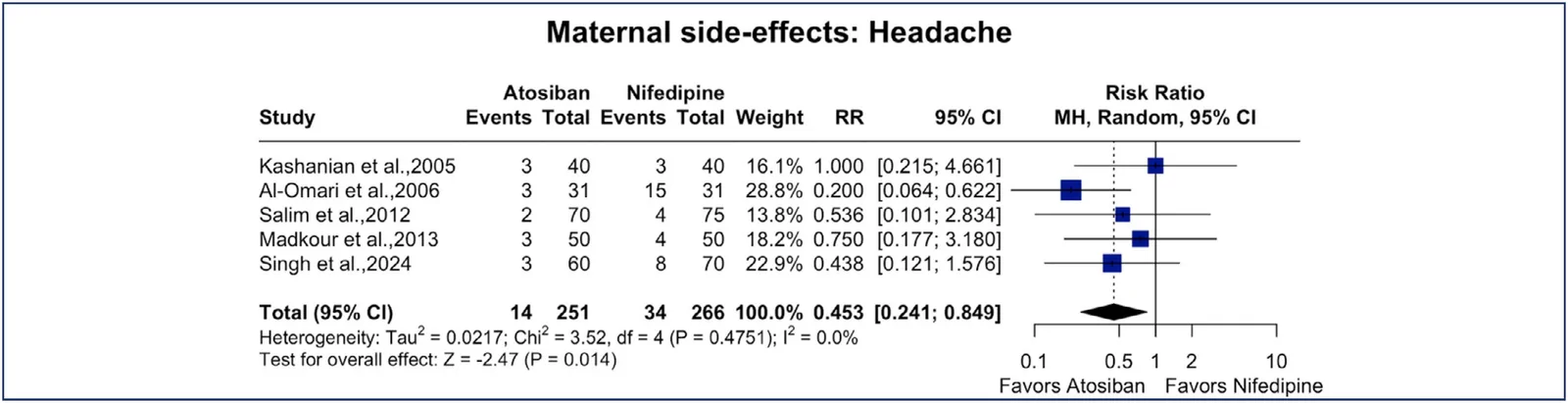
Forest plot showing pooled risk ratios for maternal headache as an adverse effect of tocolytic therapy

**Figure 5:**
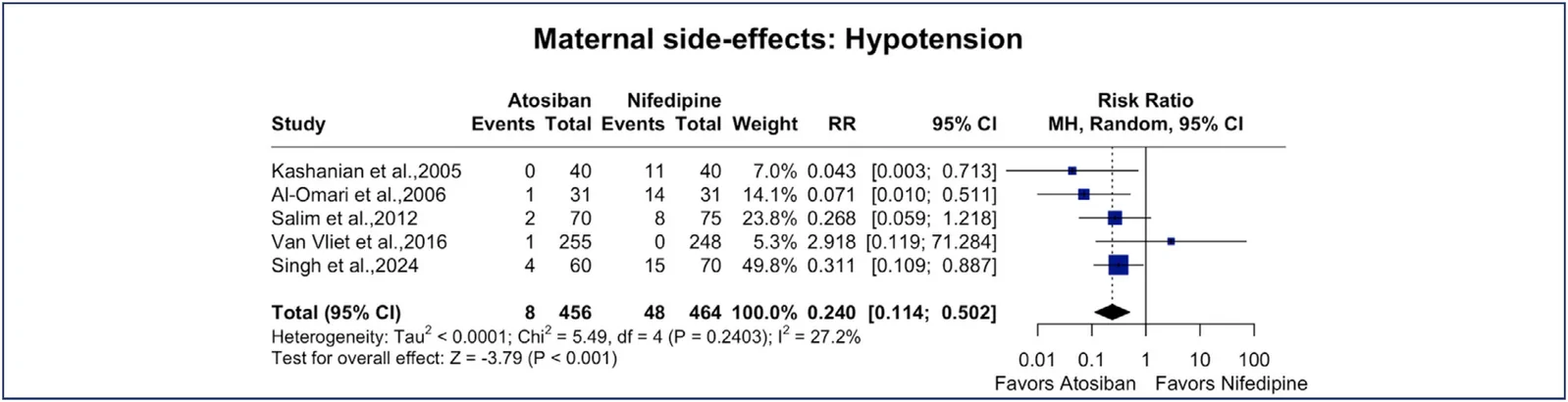
Forest plot showing pooled risk ratios for maternal hypotension as an adverse effect of tocolytic therapy

**Figure 6:**
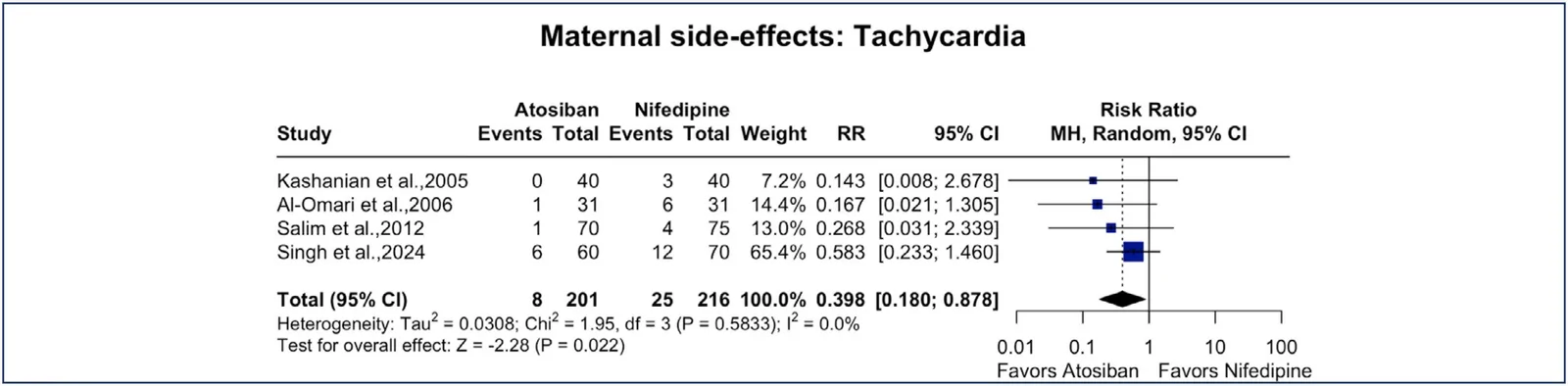
Forest plot showing pooled risk ratios for maternal tachycardia as an adverse effect of tocolytic therapy

**Figure 7:**
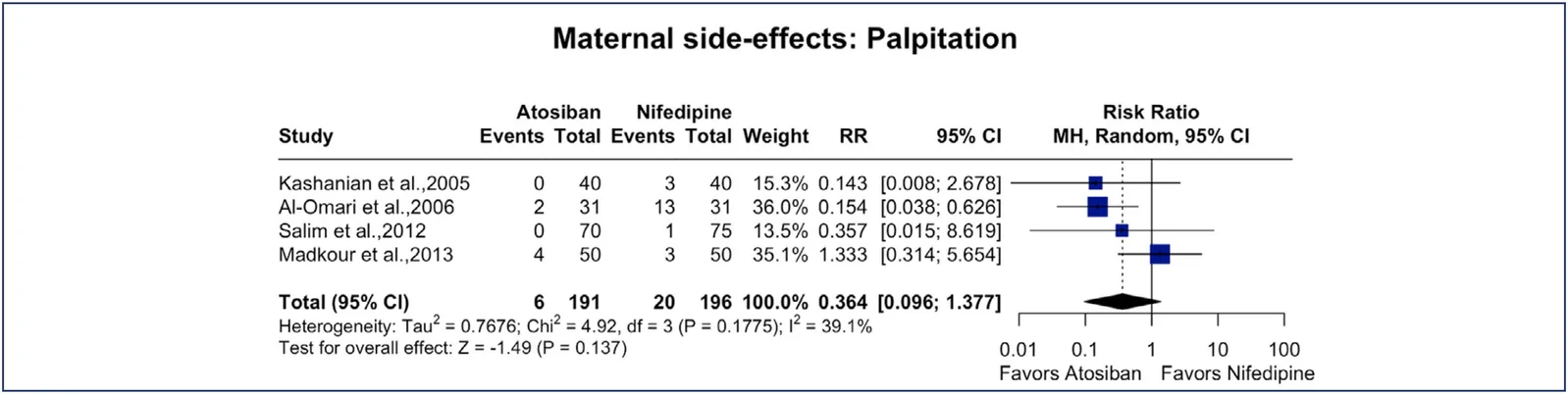
Forest plot showing pooled risk ratios for maternal palpitations as an adverse effect of tocolytic therapy.

### Neonatal outcomes

Regarding NICU admission, pooled data from two RCTs^(20,21)^ (780 neonates: 385 atosiban, 395 nifedipine) showed admission rates of 59.2% (228/385) and 47.9% (184/395) in the atosiban and nifedipine groups, respectively. This corresponds to a non-statistically significant increase in NICU admission among neonates exposed to atosiban (RR 1.36; 95% CI 0.965-1.936; p=0.079), with moderate heterogeneity (I^2^=69.8%); given the limited number of studies, this heterogeneity estimate should be interpreted with caution. (Figure 8).

**Figure 8:**
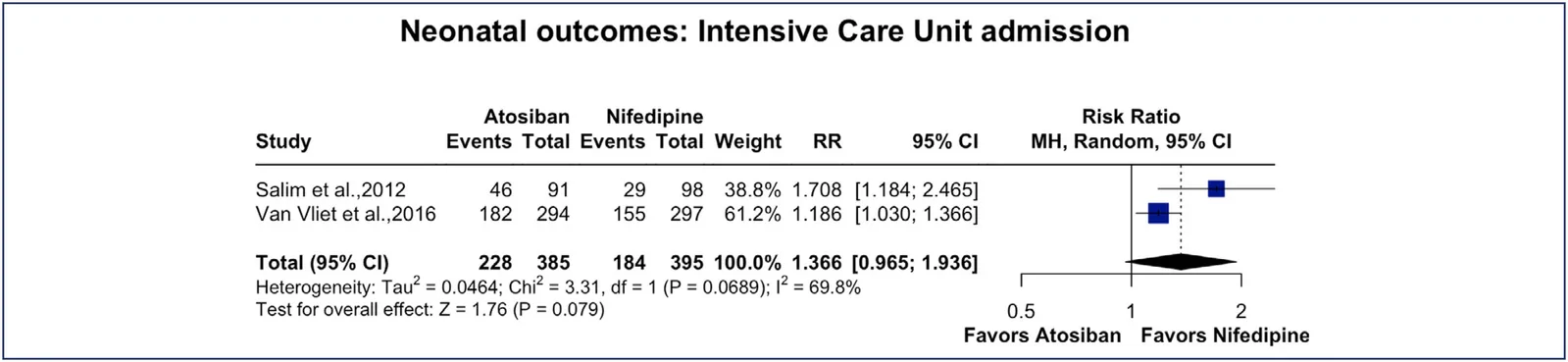
Forest plot showing pooled risk ratios for neonatal intensive care unit (NICU) admission

Neonatal sepsis (three studies,^(19-21)^ n=828) occurred in 7.1% (29/410) of neonates in the atosiban group and 7.2% (30/418) in the nifedipine group, with no significant difference (RR 0.98; 95% CI 0.60-1.60; p=0.928; I^2^=0%) (Figure 9). Hemorrhagic disease (three trials,^(19-21)^ n=828) was reported in 1.7% (7/410) with atosiban and 2.2% (9/418) with nifedipine (RR 0.79; 95% CI 0.24-2.55; p=0.691; I^2^=10.5%) (Figure 10). Neonatal apnea rates were 4.9% (27/410) and 5.5% (23/418), respectively (RR 1.15; 95% CI 0.61-2.17; p=0.66; I^2^=0%) (Figure 11). Neonatal death occurred in 3.2% (13/410) of the atosiban group and 5.0% (21/418) in the nifedipine group, with no statistically significant difference (RR 0.67; 95% CI 0.27-1.63; p=0.374; I^2^=42.5%) (Figure 12).

**Figure 9:**
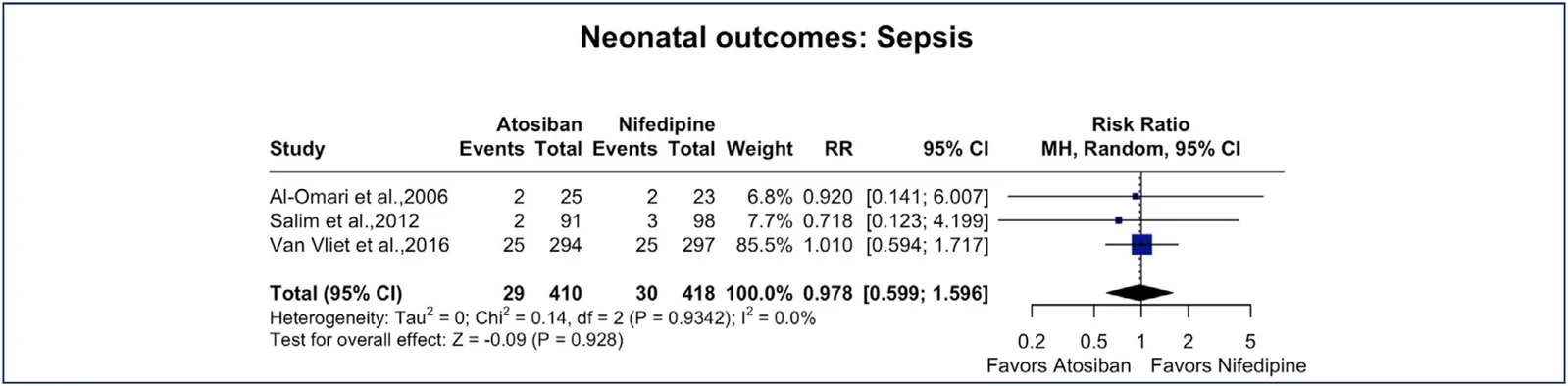
Forest plot showing pooled risk ratios for neonatal sepsis

**Figure 10:**
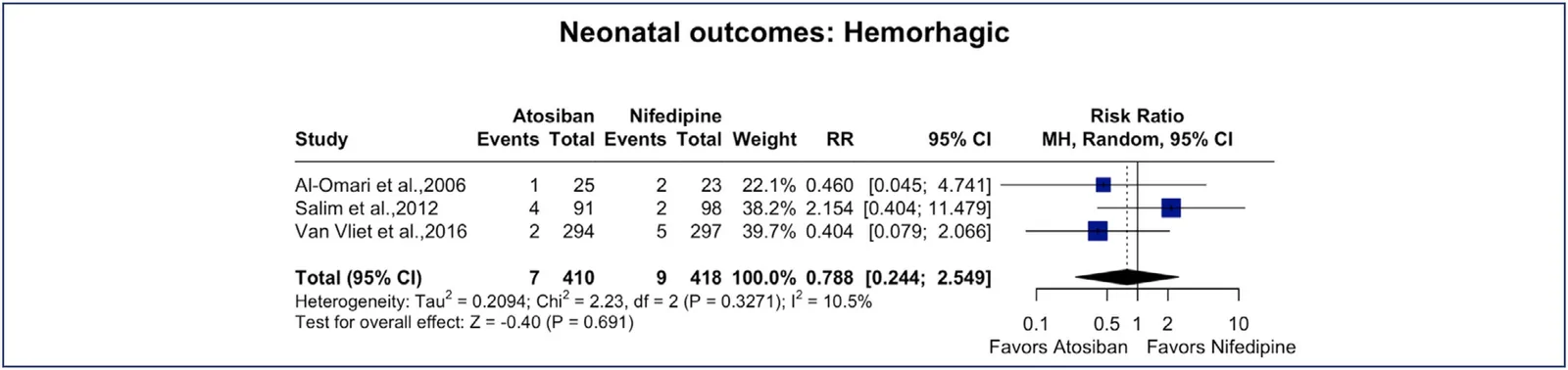
Forest plot showing pooled risk ratios for neonatal hemorrhagic disease

**Figure 11:**
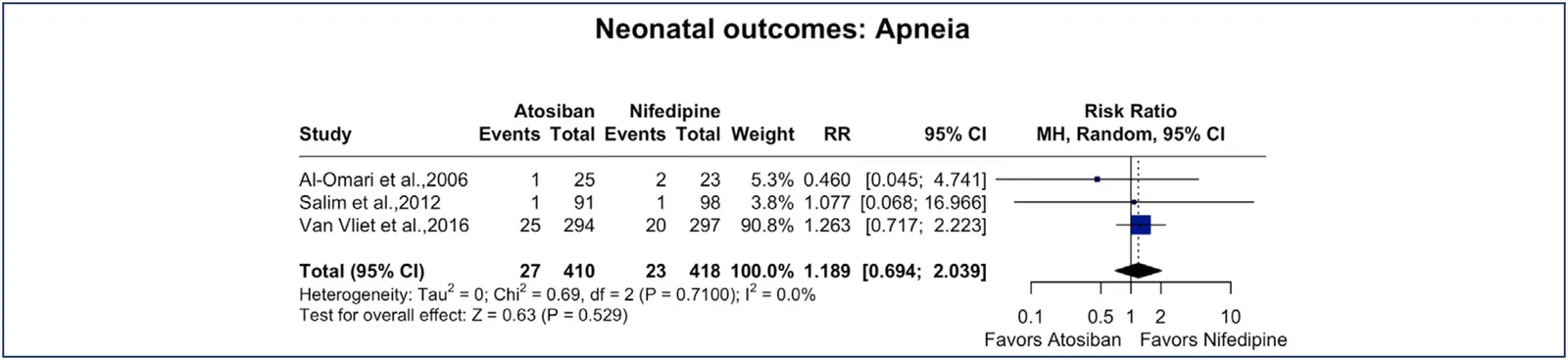
Forest plot showing pooled risk ratios for neonatal apnea

**Figure 12:**
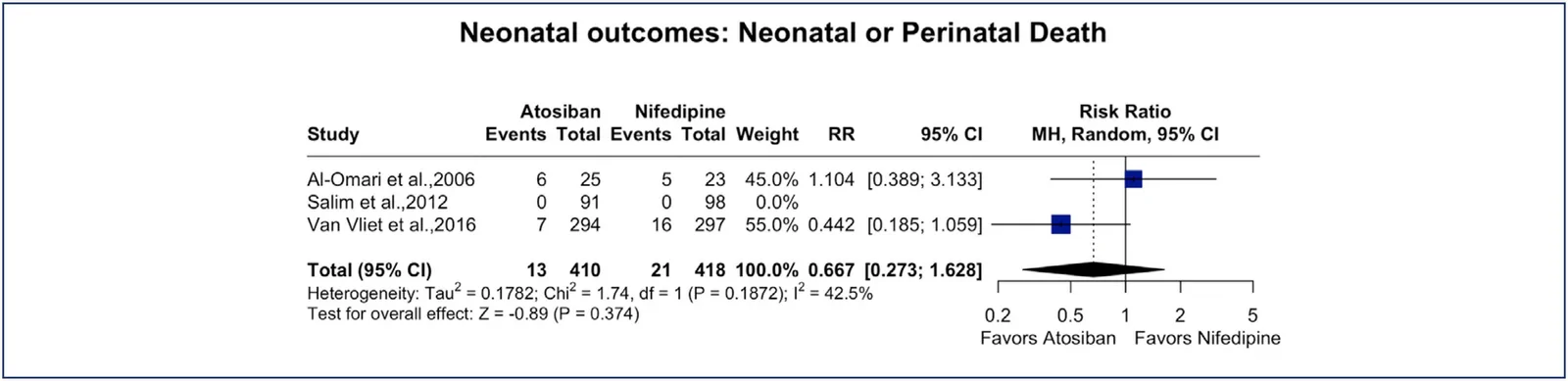
Forest plot showing pooled risk ratios for neonatal/perinatal death

**Figure 13:**
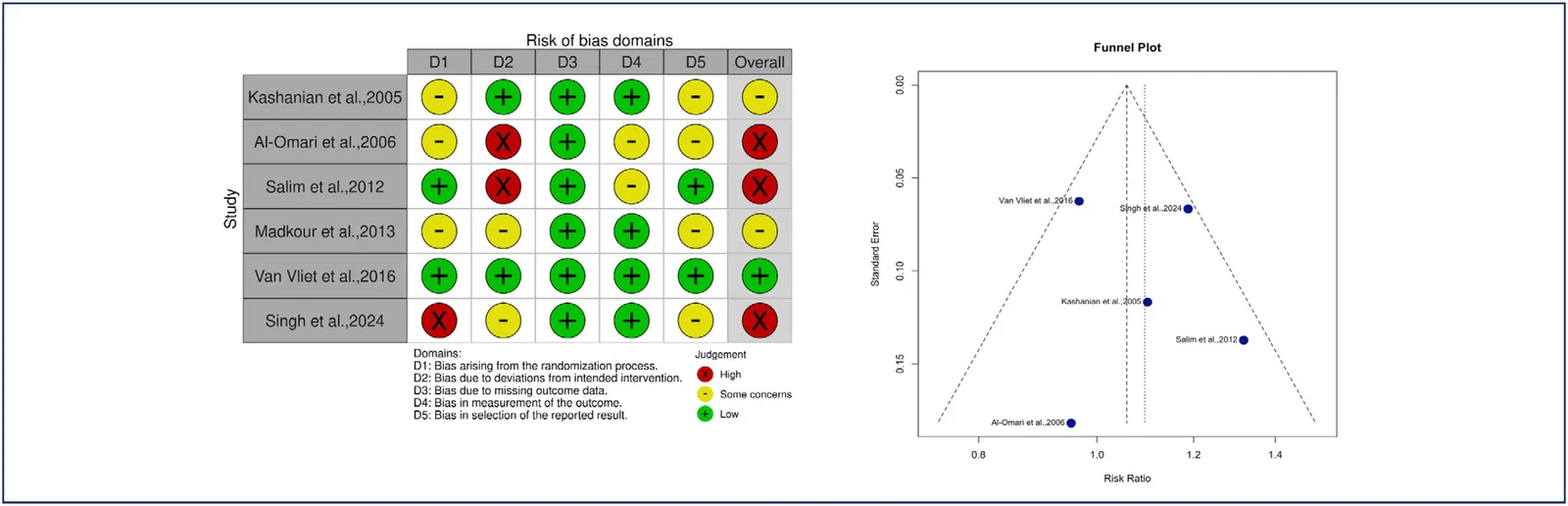
Risk-of-bias and publication bias assessment for the primary efficacy outcome (delay of delivery ≥48 hours). (A) Risk-of-bias traffic-light plot using the Cochrane RoB 2 tool across five domains: D1, bias arising from the randomization process; D2, bias due to deviations from intended interventions; D3, bias due to missing outcome data; D4, bias in measurement of the outcome; D5, bias in selection of the reported result. (B) Funnel plot assessing potential publication bias among the five trials contributing data to this outcome. Formal statistical testing (Egger's regression) was not performed given the small number of studies; visual asymmetry should therefore be interpreted with caution.

### Risk of bias and sensitivity analysis

One RCT was deemed at low overall risk of bias,^(21)^ two raised some concerns,^(18,22)^ and three were classified as high risk, largely attributable to performance bias inherent in the open-label design necessitated by differing administration routes.^(14,19,20)^

### Certainty of evidence

The certainty of evidence was assessed using the Grading of Recommendations Assessment, Development and Evaluation (GRADE) framework across all pooled outcomes. Delay of delivery ≥48 hours (primary outcome): LOW certainty, downgraded for risk of bias (three trials at high risk; open-label design) and imprecision (CI spans both clinically meaningful benefit and harm). Delay of delivery ≥7 days: LOW certainty, downgraded for risk of bias and imprecision (wide CI; point estimate near 1.0). Maternal headache: MODERATE certainty, significant and consistent reduction with atosiban (I^2^=0%), downgraded one level for risk of bias. Maternal hypotension: MODERATE certainty, significant reduction with atosiban (I^2^=27%), large effect size (RR 0.24), downgraded for risk of bias. Maternal tachycardia: MODERATE certainty, significant reduction with atosiban (I^2^=0%), downgraded for risk of bias. Maternal palpitations: LOW certainty, non-significant effect, downgraded for risk of bias and imprecision. NICU admission: VERY LOW certainty, non-statistically significant trend toward increased risk with atosiban (RR 1.36; 95% CI 0.965-1.936; p=0.079), downgraded for risk of bias, serious inconsistency (I^2^=69.8%) between the only two contributing trials; given the limited number of trials, this signal should be interpreted with caution and warrants confirmation in future studies. Neonatal death, sepsis, hemorrhagic disease, and apnea: VERY LOW certainty for each, based on rare events, wide confidence intervals, and only three trials per outcome; no conclusions regarding superiority of either agent for these outcomes are warranted.

## Discussion

Our pooled analysis establishes that atosiban and nifedipine confer comparable tocolytic efficacy, with no statistically significant difference in the proportion of women achieving delivery delay at either 48 hours or 7 days. This finding of broad equivalence is consistent with individual RCTs within our review, including Kashanian et al. (2005)^(18)^, Al-Omari et al. (2006)^(19)^, and the APOSTEL III trial,^(21)^ as well as with the prior meta-analysis by Ali et al. (2019).^(13)^ The moderate heterogeneity observed for 48-hour efficacy (I^2^=48.5%) reflects genuine clinical variability, including differences in population characteristics, gestational age ranges, and how treatment failure was defined across trials. Salim et al. (2012)^(20)^ and Singh et al. (2024)^(14)^ both reported superior 48-hour success rates with atosiban under specific study conditions, underscoring that certain clinical or demographic subgroups may respond differently, even when aggregate efficacy appears equivalent.

The clinical decision between these two agents extends well beyond tocolytic efficacy alone. Nifedipine offers substantial advantages in cost and ease of administration; the Dutch APOSTEL group demonstrated that overall healthcare costs per patient were approximately €8,500 lower with nifedipine than with atosiban.^(24)^ Conversely, atosiban may be preferred in women with underlying cardiovascular conditions or multiple gestations, where the hemodynamic consequences of nifedipine are of particular concern.^(18)^ For 7-day delay, the overall evidence supports equipoise, though Salim et al. noted a significantly higher proportion of women remaining undelivered at 7 days in the nifedipine group among singleton pregnancies beyond 28 weeks of gestation,^(20)^ a finding that warrants consideration when counseling patients about expectations for longer-duration tocolysis.

A clear divergence between the two agents emerges when examining maternal safety. Atosiban was associated with significantly lower risks of headache, hypotension, and tachycardia, with findings generally consistent across studies and low heterogeneity for these endpoints. These conclusions align with the meta-analysis by Ali et al. (2019),^(13)^ and our analysis additionally strengthens the evidence regarding hypotension reduction, for which the prior review had reported only a non-significant trend. For maternal palpitations, the point estimate favored atosiban but did not reach statistical significance, consistent with the APOSTEL III observation of numerically higher drug discontinuation rates due to side effects with nifedipine without a significant difference in composite adverse events.^(21)^ In clinical practice, this more favorable tolerability profile with atosiban is not merely a statistical distinction; it has direct implications for patient compliance, therapeutic experience, and safety in women predisposed to hemodynamic instability.

A directionally consistent, though not statistically significant, signal from this meta-analysis was a higher point estimate for NICU admission with atosiban compared with nifedipine (RR 1.36; 95% CI 0.965–1.936; p=0.079), based on the two contributing RCTs.^(20,21)^ This estimate showed substantial heterogeneity (I^2^=69.8%); given that only two trials contributed data, both the heterogeneity and the pooled estimate itself should be interpreted with caution. Despite not reaching statistical significance, both trials pointed in the same direction, and this pattern is broadly consistent with a more recent, non-randomized prospective study by Gaikwad et al. (2025)^(25)^, though not meeting RCT eligibility criteria and therefore excluded from the quantitative synthesis, reported a similarly higher NICU admission rate with atosiban (44.4% vs. 31.1%; p=0.269); notably, the study authors attributed part of this difference to confounding by indication, as atosiban was preferentially used in more clinically severe, earlier-gestational-age cases in their non-randomized cohort.

For other neonatal endpoints, including death, sepsis, hemorrhagic disease, and apnea, no statistically significant differences were identified between the two groups. While the point estimate for neonatal death numerically favored atosiban in our analysis, the confidence intervals were wide, reflecting the limited statistical power to detect differences in relatively rare events with the number of studies available.

Several limitations of this meta-analysis should be acknowledged. The review synthesizes only six trials, constraining the power to detect modest effect sizes and limiting the reliability of publication bias assessment. Moderate heterogeneity for the primary efficacy outcome and for NICU admission likely reflects true clinical diversity across study populations and settings. The open-label design of most included trials introduces risk of performance bias, though this is inherent in comparing agents with fundamentally different administration routes and is unlikely to substantially influence objective outcomes such as delivery timing. Longer-term neonatal follow-up data, including neurodevelopmental outcomes, were not available in any included trial. These limitations reinforce the need for future large-scale, standardized trials with prospective neonatal outcome ascertainment and health-economic analyses that account for differential NICU utilization.

## Conclusion

Atosiban and nifedipine offer comparable efficacy in delaying preterm birth for 48 hours and 7 days. Their profiles diverge significantly with respect to maternal safety, with atosiban carrying a demonstrably more favorable tolerability profile, including reduced risks of headache, hypotension, and tachycardia. A directionally consistent, though non-statistically significant, trend toward higher NICU admission among neonates in the atosiban group was also observed (RR 1.36; 95% CI 0.965-1.936); based on only two contributing RCTs with substantial heterogeneity, this signal should be regarded as hypothesis-generating rather than conclusive, and warrants confirmation in larger, adequately powered trials. Tocolytic selection should therefore be individualized: atosiban may be preferable for women with cardiovascular comorbidities or heightened susceptibility to nifedipine's hemodynamic effects, while nifedipine's oral route, lower cost, and possible—though not yet confirmed—neonatal resource advantage may favor its use in other contexts. Shared decision-making, with explicit communication of these trade-offs and the current uncertainty around the neonatal signal, is essential.

## Data Availability

Extracted data and R scripts for statistical analysis and meta-analysis plots have been deposited in Mendeley Data and are accessible at http://dx.doi.org/10.17632/xdbgb4wjfr.1.
