# Supplementary material for: Atosiban versus nifedipine for the management of preterm labor: a systematic review and meta-analysis of randomized controlled trials
Source: Rev Bras Ginecol Obstet. 2026 Aug 20;48:e-rbgo55. doi: 10.61622/rbgo/2026rbgo55 (PMC13690373; doi:10.61622/rbgo/2026rbgo55)
Supplement: Supplementary file 1 [file 1806-9339-rbgo-48-e-rbgo55-Supp01.pdf]

Supplementary Appendix 1 — Search Strategy

Search conducted on May 25, 2025. Total results: 201 records. After deduplication: 110 unique records screened (91 duplicates removed). No language restrictions were applied. The ICTRP search yielded 0 records. Manual screening of reference lists of included studies, prior systematic reviews, and relevant meta-analyses was also performed but did not incorporated any new RCT to our literature search.

Supplementary Table S1. Complete search strings by database.

Platform	Search String	Results
PubMed	("nifedipine"[MeSH Terms] OR nifedipine[tiab] OR Cordipin[tiab] OR Corinfar[tiab] OR Nifangin[tiab] OR Procardia[tiab] OR fenigidin[tiab] OR Vascard[tiab] OR Adalat[tiab]) AND ("atosiban"[Supplementary Concept] OR atosiban[tiab] OR Tractocile[tiab] OR Oxytocin Receptor Antagonists[MeSH Terms] OR oxytocin receptor antagonist*[tiab]) AND ("obstetric labor, premature"[MeSH Terms] OR "Premature Birth"[MeSH Terms] OR preterm labor[tiab] OR preterm birth[tiab] OR preterm delivery[tiab] OR premature labor[tiab] OR premature obstetric labor[tiab] OR early uterine contraction[tiab] OR premature birth[tiab] OR premature labour[tiab] OR preterm labour[tiab])	78
Cochrane Library	(nifedipine OR Cordipin OR Corinfar OR Nifangin OR Procardia OR fenigidin OR Vascard OR Adalat) AND (atosiban OR Tractocile OR (oxytocin NEXT receptor NEXT antagonist*) OR (oxytocin NEXT antagonist*)) AND ("preterm labor" OR "preterm labour" OR "preterm birth" OR "preterm delivery" OR "premature labor" OR "premature labour" OR "premature birth" OR "premature delivery" OR "obstetric labor, premature" OR "obstetric labour, premature" OR (early NEXT uterine NEXT contraction*))	36
EBSCOHost	(nifedipine OR Cordipin OR Corinfar OR Nifangin OR Procardia OR fenigidin OR Vascard OR Adalat) AND (atosiban OR Tractocile OR "oxytocin receptor antagonist*" OR "oxytocin antagonist*") AND ("preterm labor" OR "preterm labour" OR "preterm birth" OR "preterm delivery" OR "premature labor" OR "premature labour" OR "premature birth" OR "premature delivery" OR "obstetric labor, premature" OR "obstetric labour, premature" OR "early uterine contraction*")	82
ClinicalTrials.gov	(nifedipine OR Cordipin OR Corinfar OR Nifangin OR Procardia OR fenigidin OR Vascard OR Adalat) AND (atosiban OR Tractocile OR oxytocin receptor antagonist OR oxytocin antagonist) AND (preterm labor OR preterm labour OR preterm birth OR preterm delivery OR premature labor OR premature labour OR premature birth OR premature delivery OR obstetric labor premature OR obstetric labour premature OR early uterine contraction)	5
ICTRP	(nifedipine OR Cordipin OR Corinfar OR Nifangin OR Procardia OR fenigidin OR Vascard OR Adalat) AND (atosiban OR Tractocile OR oxytocin receptor antagonist OR oxytocin antagonist) AND (preterm labor OR preterm labour OR preterm birth OR preterm delivery OR premature labor OR premature labour OR premature birth OR premature delivery OR obstetric labor premature OR obstetric labour premature OR early uterine contraction)	0

ICTRP: International Clinical Trials Registry Platform.

Supplementary Appendix 2 — Study Characteristics

Supplementary Table S2. Characteristics of included studies.

Study (Author, Year, Country)	Population	Atosiban Regimen	Nifedipine Regimen	Design	Age (Mean ± SD)	Prior Preterm Birth (%)	Gestational Age (Mean ± SD)	Parity	Prior Tocolysis	Cervical Dilation
Kashanian et al., 2005; Iran	GA 26-34 weeks; ≥4 contractions/20 min or ≥8/60 min; dilation ≥1 cm; effacement ≥50%	300 μg/min IV; max 12h or 6h after contraction cessation	10 mg SL q20 min ×4, then 20 mg PO q6h ×24h, then q8h ×24h, then 10 mg q8h ×24h	RCT	N/A	N/A	N/A	N/A	N/A	N/A
Al-Omari et al., 2006; Iraq	Women 18-35 years; GA 24-35 weeks; PTL per Moutquin criteria	6.75 mg IV bolus, then 18 mg/h IV ×3h, then 6 mg/h for 24-48h	10 mg PO q15 min until quiescence (max 40 mg 1st hour), then 10 mg q4-6h	RCT	27.94 ± 6.32 (N) 27.45 ± 5.93 (A)	N/A	31.19 ± 2.82 (N) 30.84 ± 2.86 (A)	N: 25% nulliparous, 75% multiparous A: 35.5% nulliparous, 25% multiparous	N/A	N: <1 cm 59.4%, 2 cm 31.3%, 3 cm 9.3% A: <1 cm 67.7%, 2 cm 19.4%, 3 cm 12.9%
Madkour et al., 2013; UAE	Women 20-35 years; singleton; GA 26-34 weeks; regular contractions; dilation 0-3 cm; effacement ≥50%	6.75 mg IV bolus, 300 τ g/min IV ×3h, then 100 τ g/min for 48-96h	20 mg PO, then 20 mg after 30 min, then 20 mg q3-8h for 48-72h (max 160 mg/d)	RCT	28.1 (N) 31.3 (A)	N/A	N/A	N: 38 primigravida, 12 para 2 A: 40 primigravida, 9 para 1, 1 para 3	N/A	N/A
Salim et al., 2012; Israel	Women with PTL; intact membranes; GA 24+0-33+6 weeks	6.75 mg IV bolus, 300 τ g/min IV ×3h, then 100 τ g/min for 45h	20 mg PO loading, then up to two 20 mg doses q20-30 min, then 20-40 mg PO qid ×48h	RCT	27 (19-48) (N) 28 (20-44) (A)	N: 0 prior 84.0%, ≥1 prior 16.0% A: 0 prior 78.6%, ≥1 prior 21.4%	31.8 (25.0-33.8) (N) 31.1 (24.1-33.8) (A)	N: 49.3% nulliparous A: 35.7% nulliparous	N/A	N/R
Van Vliet et al., 2016; Netherlands (APOSTEL III)	Women ≥18 years; threatened PTL; GA 25+0-34+0 weeks; singleton or multiple gestation	6.75 mg IV bolus, then 18 mg/h IV ×3h, then 6 mg/h for 45h	20 mg PO, then 20 mg slow-release PO q6h ×47h	Multicenter RCT	30.7 (26.2-34.0) (N) 30.2 (27.2-33.0) (A)	N: 13% (33/255) A: 12% (30/255)	30.3 (28.4-32.1) (N) 30.3 (28.1-31.7) (A)	N: 65% nulliparous A: 67% nulliparous	N: 34% (85/248) A: 35% (88/255)	N/A
Singh et al., 2024; India	Women >18 years; threatened PTL; GA 25-34 weeks; dilation >1 and <3 cm; intact membranes; ≥4 contractions/20 min or ≥8/60 min; effacement >80%	6.75 mg IV bolus, then 300 τ g/min IV ×3h, then 100 τ g/min up to 45h	20 mg PO, max 40 mg 1st hour, max 100-120 mg total	RCT	N/A	N/A	N/A	N/A	N/A	N/A

Abbreviations: A, atosiban group; GA, gestational age; ICTRP, International Clinical Trials Registry Platform; IV, intravenous; N, nifedipine group; N/A, not available; N/R, not reported; PO, oral; PTL, preterm labor; qid, four times daily; RCT, randomized controlled trial; SD, standard deviation; SL, sublingual.
